# Omega-3 and omega-6 fatty acid differentially impact cardiolipin remodeling in activated macrophage

**DOI:** 10.1186/s12944-018-0845-y

**Published:** 2018-08-28

**Authors:** Wan-Hsin Chang, Hsiu-Chi Ting, Wei-Wei Chen, Jui-Fen Chan, Yuan-Hao Howard Hsu

**Affiliations:** 1 Department of Chemistry, Tunghai University, Taichung, No.1727, Sec4, Taiwan Boulevard, Xitun District, Taichung, 40704 Taiwan, Republic of China; 2 Life Science Research Center, Tunghai University, Taichung, No.1727, Sec4, Taiwan Boulevard, Xitun District, Taichung, 40704 Taiwan, Republic of China

**Keywords:** Macrophage, Cardiolipin, Monolysocardiolipin, Polyunsaturated fatty acid, Inflammation, Liquid chromatography-mass spectrometry

## Abstract

**Background:**

The macrophage plays an important role in innate immunity to induce immune responses. Lipid replacement therapy has been shown to change the lipid compositions of mitochondria and potentially becomes an alternative to reduce the inflammatory response.

**Methods:**

We examined the effects of omega-6 arachidonic acid (AA), omega-3 eicosapentaenoic acid (EPA), and omega-3 docosahexaenoic acid (DHA) supplementation on the activated the macrophage cell line RAW264.7 via KdO_2_-lipid A (KLA). The mitochondrial cardiolipin (CL) and monolysocardiolipin (MLCL) were analyzed by LC-MS.

**Results:**

After macrophage activation by KLA, CL shifted to saturated species, but did not affect the quantity of CL. Inhibition of delta 6 desaturase also resulted in the same trend of CL species shift. We further examined the changes in CL and MLCL species induced by polyunsaturated fatty acid supplementation during inflammation. After supplementation of AA, EPA and DHA, the MLCL/CL ratio increased significantly in all treatments. The percentages of the long-chain species highly elevated and those of short-chain species reduced in both CL and MLCL.

**Conclusions:**

Comparisons of AA, EPA and DHA supplementation revealed that the 20-carbon EPA (20:5) and AA (20:4) triggered higher incorporation and CL remodeling efficiency than 22-carbon DHA (22:6). EPA supplementation not only efficiently extended the chain length of CL but also increased the unsaturation of CL.

**Electronic supplementary material:**

The online version of this article (10.1186/s12944-018-0845-y) contains supplementary material, which is available to authorized users.

## Background

Omega-3 fatty acids, docosahexaenoic acid (DHA) and eicosapentaenoic acid (EPA) are common supplements in the diet. A plethora of studies have elucidated their roles in anti-inflammation and health benefits [1]. While DHA and EPA are upstream molecules for pre-resolving lipid mediator synthesis, such as resolvins, protectins, and maresins [2], arachidonic acid (AA) is primarily further metabolized into eicosanoids, which are recognized as inflammatory lipid mediators [3]. Studies have reported about dietary fatty acid incorporation into phospholipids in humans and CL in both animal and cell models [4–7]. Polyunsaturated fatty acids (PUFAs) can be incorporated in CL acyl contents, including DHA, EPA and AA, and other fatty acids contain more than one double bond, which are known as *n*-3 and *n*-6 PUFAs [6].

CL, a mitochondrial phospholipid containing four fatty acyl chains, is localized restrictedly in the inner membrane and regulates mitochondrial physiological functions. In CL biosynthesis, phosphatidic acid is metabolized into phosphatidylglycerol that combines with CDP-diacylglycerol by CL synthase to form immature CL, which is further remodeled by transacylase and/or acyltransferase by which various fatty acyl chains are incorporated [8]. Monolysocardiolipin (MLCL) could be generated through deacylation by phospholipases A_2_s (PLA_2_s) or transacylation between CL and lysophospholipid by transacylase activity of tafazzin [9, 10]. A decrease in CL together with an increase in MLCL was found in fibroblasts from patients with Barth syndrome [11] and the imbalanced CL species is related to the etiology of this genetic disorder [12].

Mitochondria are known for their pivotal roles in energy production, apoptosis, calcium buffering, and metabolic intermediate support. In addition to the important roles in the aforementioned processes, the link between mitochondria and inflammation has been studied due to the association of mitochondrial dysfunction with several acute and chronic inflammations [13]. Imbalanced redox status in mitochondria induces excess reactive oxygen species (ROS) generation, and several studies have also shown that mitochondrial ROS triggers the production of proinflammatory cytokines in cells, such as IL-6 and TNF [14]. Mitochondrial ROS also mediates inflammation via pyrin domain–containing protein 3 (NLRP3) inflammasome activation, leading to matured IL-1β production and considered as an ROS-dependent pathway for NLRP3 inflammasome activation [15].

*n*-3 PUFA and *n*-6 PUFA that are incorporated into mitochondria also have higher propensities to be attacked by ROS [16], along with the important role of CL in inflammasome activation and the liberation of lipid mediators. The liberation of oxygenated PUFAs contributes to a mitochondrial-mediated production of oxygenated fatty acids, which can function as lipid mediators in inflammatory responses [17]. Fatty acyl content in CL is therefore considered to play a key role in CL pathophysiology. Recent evidence also indicates that mitochondrial cardiolipin (CL) externalization activates NLRP3 inflammasome, which demonstrates the existence of an ROS-independent pathway for NLRP3 inflammasome activation [18, 19]. These studies evidently reveal the close relationships among inflammation, mitochondria, and their unique lipid component–CL.

A previous study has shown the synergic effects of ATP and KdO_2_-lipid A (KLA) in mouse bone-marrow macrophages involved in the activation of pro-inflammatory and anti-inflammatory cytokines, JAK-STAT pathway, cell cycle, and apoptosis signaling pathway, along with the metabolism of eicosanoids, sphingolipids, and sterols [20]. The anti-inflammatory and inflammatory characteristics of *n*-3 and *n*-6 PUFAs have also been investigated [21], however, how do these two types of PUFA differentially affect the mitochondrial CL, is not yet clear. In this study, we focused on CL metabolism in KLA-activated RAW264.7 cells, combined with the effect of PUFA supplementation on CL. We used RAW264.7 macrophage to study the differential effects of *n*-3 and *n*-6 PUFA treatments on CL and MLCL remodeling during KLA induced inflammation. CL and MLCL were extracted and monitored by HPLC-ion trap mass spectrometer to investigate the PUFA treatment effects on cardiolipin in the activated macrophage.

## Methods

### Material

DHA, EPA, AA, formic acid, and ammonium formate was purchased from Sigma-Aldrich, USA. Acetonitrile (ACN) and chloroform was purchased from ECHO Chemical, Taiwan. Methanol was bought from MACRON, USA. 2-propanol (IPA) was bought from J.T. Baker, USA. Fatty acid free bovine serum albumin was purchased from Akron, USA. Dimethyl sulfoxide was acquired from Bio Basic Inc., Canada. Dulbecco’s modified eagle medium, fetal bovine serum, penicillin-streptomycin and 25 mM HEPES for cell culture were from Gibco, USA. CL standard (C14:0)_4_ was from Avanti, USA. Kdo_2_-Lipid A (KLA) was purchased from Cayman. SC 26196 (∆6 desaturase inhibitor) was purchased from Tocris Bioscience, UK.

### Cell culture

Raw 264.7 cell was kindly provided by Dr. Wei-Hao Peng. Before cell cultivation, FBS was heated in 57°C water bath to inactivate complement system, preventing unexpected activation of the cell. The cells were cultured in Dulbecco’s modified eagle medium with heated 10% fetal bovine serum, 50 Unit/ml of penicillin, 50 μg/ml of streptomycin and 25 mM HEPES in 0.5% CO_2_ at 37 °C. The cells at 90% confluency were plated to 6-cm culture dishes in 1/35 ratio, and waited for cell attachment for 6 h. Control group was added with 100 μM BSA. The other three groups were additionally supplemented with 100 μM DHA, 100 μM EPA and 100 μM AA for 24 h. KLA was added to experimental groups and cultured for additional 24 h. In the ∆6 desaturase inhibitor experiment, 20 μM of SC 26196 (∆6 desaturase inhibitor) was added to RAW264.7 cells, which were then harvested after 24-h culture. The cells were harvested in 1 ml PBS and 100 μl was taken for protein quantification. The rest of the cells were stored in − 80 °C for lipid extraction.

### Protein quantification

Cells were lysed by RIPA buffer and sonication on ice. The deionized distilled water was added to make a final volume of 200 μl. The samples were centrifuged at 10000 rpm for 5 min, and then the supernatant was mixed with Bio-Rad protein assay dye reagent (Bio-Rad) for Bradford protein-binding assay. Absorption spectrum at 595 nm was detected for protein quantification (SpectraMax M series Multi-Mode Microplate Readers, Molecular Device).

### Lipid extraction

The total lipids of collected cells were extracted by Bligh-Dyer’s method [22] with slight modification. Briefly, the cell pallets were added 12.5 ng CL(14:0)_4_ as internal standard and were sonicated in 2 ml methanol. After sonication, final volume of 3 ml of dichloromethane/methanol (1:2) was mixed with samples and vortex for 10 min. Then, 1 ml of dichloromethane and 1 ml of DDW were added to the samples and further vortex for 10 min. The lower phase in the test tube was collected by centrifugation (3000 rpm for 5 min) and dried under nitrogen gas.

### Mass spectrometry analysis

The extracted total lipids were sonicated and dissolved in ACN/IPA/H_2_O (65:30:5) for 30 s. The samples were analyzed by LC/MS Ion-Trap (Bruker). The recipe of HPLC mobile phases are shown as following - solution A: ACN:H_2_O (60:40), 10 mM ammonium formate, 0.1% formic acid and solution B: IPA:ACN (90:10), 10 mM ammonium formate, 0.1% formic acid [23]. Gradient was from 60% solution A to 100% solution B in 25 min, maintained 100% solution B until 40 min, and then returned to 60% solution A in a *Acclaim RSLC 120* C18 2.1 mm × 150 mm 2.2 μm column (Thermo, USA) at a flow rate of 0.2 ml/min and 55 °C. Data was further analyzed by Bruker DataAnalysis (ver.3.4).

### Identification of CL and MLCL in RAW264.7 cells

The CL and MLCL species in RAW264.7 cells were identified by LC-MS/MS. Based on the structure of CL and its charge, the extracted total lipid was analyzed by reverse-phase chromatography and mass spectrometry in negative mode. The methodology for CL analysis and identification has been described previously [6, 24]. In short, the fragmentation of CL by tandem mass spectrometry determined the fatty acyl compositions of each CL species. Internal CL standard CL(14:0)_4_ was added before lipid extraction. The CL quantity was semi-quantified by the relative extract ion current (XIC) of the target CL to the XIC of the internal standard. In our measurement, RAW264.7 cells contain 55 CL species with 42 types of molecular weights, and the acyl chains of each species were identified. For MLCL analysis, the signal of extracted ion chromatogram of MLCL standard (C14:0)_3_ appeared by around 24 min in our liquid chromatography gradient program. The retention time of MLCL in samples was around 26–30 min. MLCL species showed in five clusters in m/z range of 1100–1300. There are 33 MLCL species with 16 molecular weights. All of the identification of CL and MLCL was carried out by secondary fragmentation.

### Statistical analysis

Statistical analysis was performed by Prism 5.0. All statistical analysis was performed by student t-test except multi-group comparisons were carried out using one-way ANOVA followed by Tukey test, which would be mentioned in the text. The *p*-value < 0.05 was considered statistically significant.

## Results

### RAW264.7 cell activation minor changed cardiolipin saturation

**Fig. 1:**
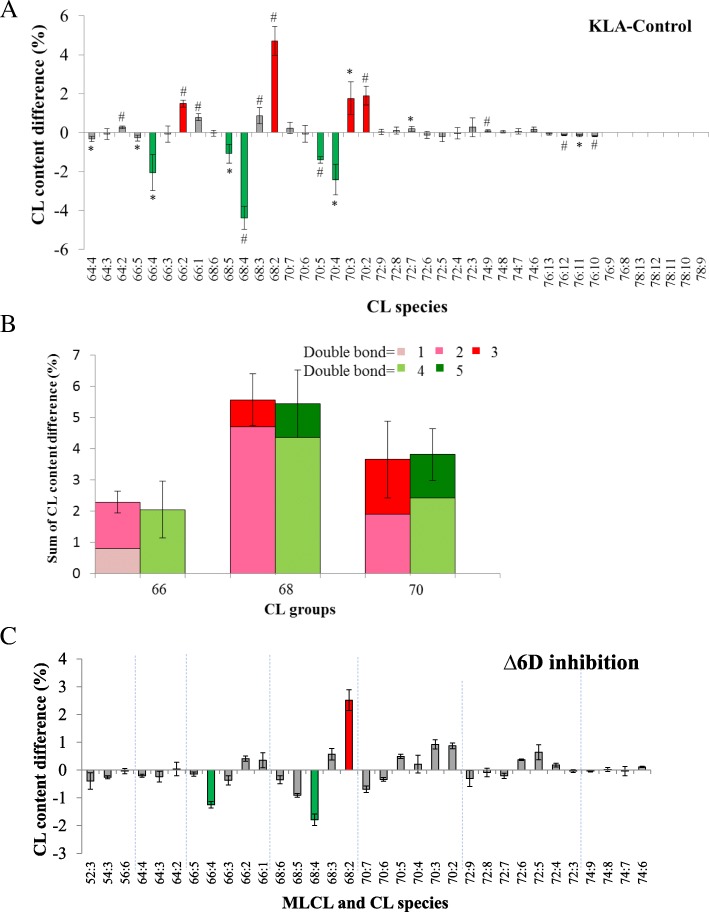
The changes of CL and MLCL species in KLA-activated RAW 264.7 cell. KLA triggered-cell is compared with inactive cell. In panel (**a**), CL species with red bar shows changes more than 1% and green bar shows changes more than − 1%, which is the one-fifth of maximal changes. In panel (**b**), The comparison of content containing different numbers of DBs in selected CL. C66, C68, and C70 are chosen, and content increase and decrease are shown in red and green. Different color means different numbers of DBs. **c** The changes of CL and MLCL species after addition of 20 μM SC 26196 (∆6 desaturase inhibitor) to RAW264.7 cells are shown. *p < 0.05,#*p*,0.01

KLA activates macrophages through Toll-like receptor 4 signaling and triggers the changes of CL saturation, which are critical for the mitochondrial function [25]. Despite the observation of these saturation changes, the quantity and the acyl chains of those CL and MLCL species have not been identified to evaluate the impact to mitochondria. We carried out mass spectrometry experiments to decipher the details of these changes. In our experiments, KLA activation of the cell increased the saturation of the fatty acyl chains on CL (Fig. 1a). We noticed that the saturation shifts were localized within the same carbon envelope. Therefore, we summed up the percent changes of the CL species containing the same number of carbon atoms (Fig. 1b). In the same CL clusters, the sums of the decreased percentages of CL containing one, two, and three DBs (CL66:2, CL66:1, CL68:3, CL68:2, CL70:3, and CL70:2) were similar to the sums of the increased total percentages of CL containing four and five DBs (CL66:4, CL68:5, CL68:4, CL70:5, and CL70:4), which indicates that the changes were within each CL cluster. This result suggested KLA triggered the inhibition of the desaturases to inhibit fatty acid desaturation and further elongation.

If these saturation shift effects were related to the inhibition of the fatty acid elongation, the crucial Δ6 desaturase for the fatty acid synthesis would also be inhibited. Therefore, RAW264.7 cells were treated with Δ6 desaturase inhibitors to inhibit Δ6 desaturase. As we expected, the similar desaturation effects on CL re-appeared (Fig. 1c). The affected CL species were also in the C66, C68, and C70 clusters. These results confirm the participation of desaturase in CL remodeling and suggest the involvement of desaturase in KLA activation. Based on the identified CL and MLCL species, the most impacted fatty acyl chains by the desaturases were saturated 16:0 and 18:0 fatty acyl chains, which can lead to a more staggered geometry of CL.

**Fig. 2:**
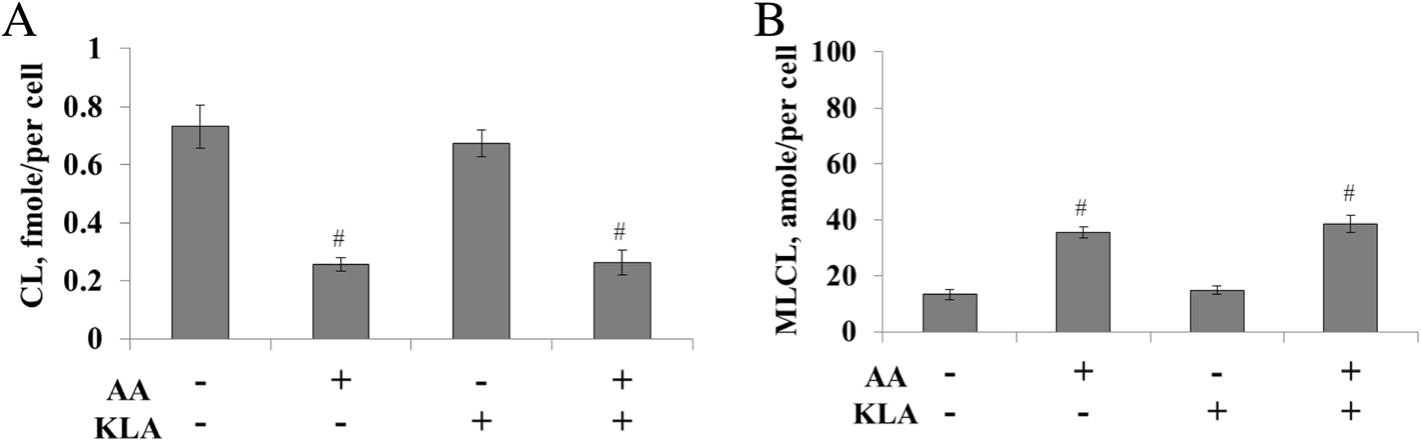
Quantification of CL and MLCL after KLA and AA treatment of RAW 264.7 cells. The total lipids of inactive and active cells were extracted and CL (**a**) and MLCL (**b**) are semi-quantified shown by amole per single cell

The quantities of CL and MLCL were also evaluated by mass spectrometry (Fig. 2). Surprisingly, the quantities of both CL and MLCL stayed the same upon KLA activation, considering the significant changes of saturation of CL. This indicated KLA activation triggered intensive CL remodeling, but did not affect the synthesis or degradation of CL.

### Omega-6 AA supplementation impaired mitochondrial lipid membrane

Although KLA activates macrophage, the production of eicosanoids is elevated after omega-6 AA, omega-3 EPA and omega-3 DHA supplementation [21, 26]. While the exogenous PUFA provides the source for eicosanoid production, the excessive PUFA may retain in the cellular membranes and change the compositions of the mitochondrial membranes. And this in turn may disturb the desaturation process of mitochondrial CL upon KLA activation.

We therefore measured the effect of *n*-6 PUFA on CL remodeling in both inactive and active RAW264.7 cells. The experiments started with supplementation of 100 μM AA to RAW264.7 cells for 24 h and then stimulated by KLA for an additional 24 h. The results showed that AA supplementation reduced 61% of CL in the active RAW264.7 cells (Fig. 2a) and surprisingly had a similar effect to the inactive cells. The reduction of CL was accompanied with the elevation of MLCL content (Fig. 2b), suggesting the hydrolysis of CL to generate MLCL. It is worth to note that not all of the disappeared CLs were converted to the increased quantities of MLCL. Further hydrolysis of the MLCL may be participated with CL degradation.

**Fig. 3:**
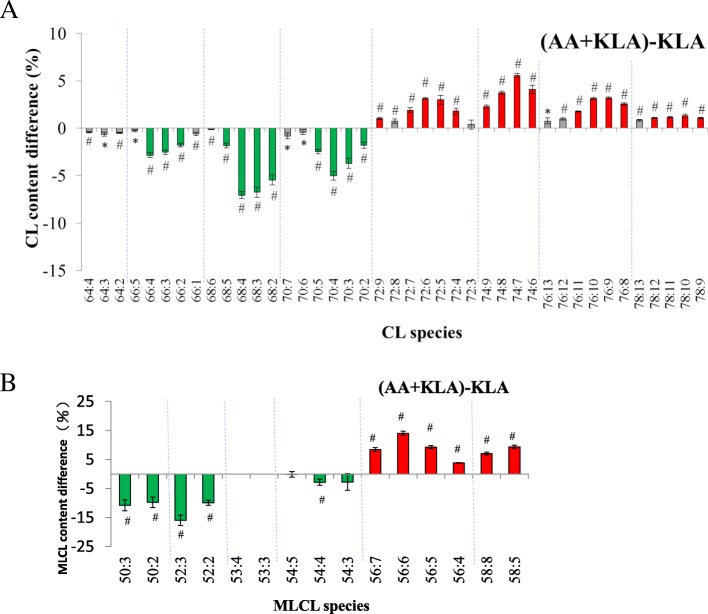
The change of contents of CL and MLCL after AA and KLA addition. RAW264.7 cells were stimulated by 100 μM AA and 100 ng/ml KLA. The CL and MLCL species difference between AA+KLA and KLA group were performed by direct deduction and shown in panel (**a**) and panel (**b**). Red represents the content difference more than 1 and 2.5% whereas green more than − 1 and − 2.5%, which is the one-fifth of the maximal difference, in CL and MLCL measurements, respectively. *p < 0.05, #*p* < 0.01

As we expected, AA supplementation changed the CL species and drastically increased the percentage of long-chain CL species (Fig. 3a). However, the supplementation also increased the highly unsaturated CL, which is a reverse effect of the KLA activation. The reduction of short-chain MLCL and the increase of long-chain MLCL were a similar trend as CL measurement in KLA-activated RAW264.7 cells (Fig. 3b). This un-favored desaturation may lead to the drop the CL and the increase of MLCL/CL ratio.

In the absence of KLA activation with AA supplementation, CL and MLCL contents showed similar trend as the KLA-activated experiments (Additional file 1). The MLCL/CL ratio increased 6.7-fold and 7.8-fold after AA supplementation in active and inactive RAW264.7 cells. The effects of AA supplementation on the CL and MLCL quantities were independent of KLA activation. We had also primed RAW264.7 cells by KLA first and then supplied AA to examine if any change occurred in the CL pattern, but no difference was observed compared to our original experimental design. Overall, AA supplementation stimulated the remodeling process to change CL species, and activates the hydrolysis of CL to MLCL. The drastic CL and MLCL changes upon AA supplementation were overwhelmingly larger than the desaturation effects by KLA activation alone.

### Omega-3 EPA and DHA elevated MLCL/CL

AA triggered CL remodeling and caused the accumulation of MLCL. The increase of MLCL/CL ratio is a reverse sign of mitochondrial function. Although EPA and DHA have been shown to produce anti-inflammatory eicosanoids to resolve inflammation and were commonly supplemented in diet, these omega-3 PUFAs may also incorporate into mitochondria and are susceptible toward oxidation as AA. We would like to evaluate the differences between omega-6 and omega-3 fatty acid supplementation.

**Fig. 4:**
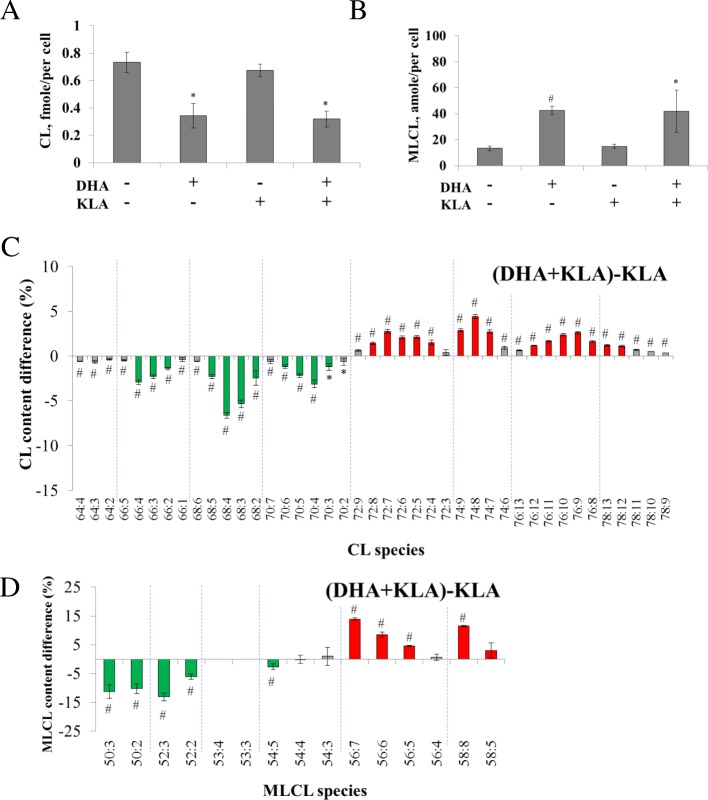
The change of amount and contents of CL and MLCL after DHA and KLA addition. RAW264.7 cells were stimulated by 100 μM DHA and 100 ng/ml KLA. The amount of CL (**a**) and MLCL (**b**) were quantified and normalized via protein quantification. The CL and MLCL species difference between AA+KLA and KLA group were performed by direct deduction and shown in panel (**c**) and panel (**d**). Red represents the content difference more than 1 and 2.5% whereas green represents more than − 1 and − 2.5%, which is the one-fifth of the maximal difference, in CL and MLCL measurements, respectively. *p < 0.05, #*p* < 0.01

We supplemented ω-3 DHA and EPA to RAW264.7 cells. A similar experimental strategy was performed as that with AA treatment. The results showed that DHA supplementation reduced 53% of CL content (Fig. 4a), decreased short-chain CL, and increased long-chain CL (Fig. 4c) in KLA-activated RAW264.7 cells. Furthermore, DHA induced a 3-fold increase of MLCL content (Fig. 4b), a decrease of short-chain MLCL, and an increase of long-chain MLCL in KLA-activated RAW264.7 cells (Fig. 4d). The MLCL/CL ratio increased 6.0 and 6.8 fold after DHA supplementation in active and inactive RAW264.7 cells. In accordance with AA treatment, CL and MLCL contents showed similar trends when DHA was supplemented in the presence and absence of KLA activation (Additional file 2).

**Fig. 5:**
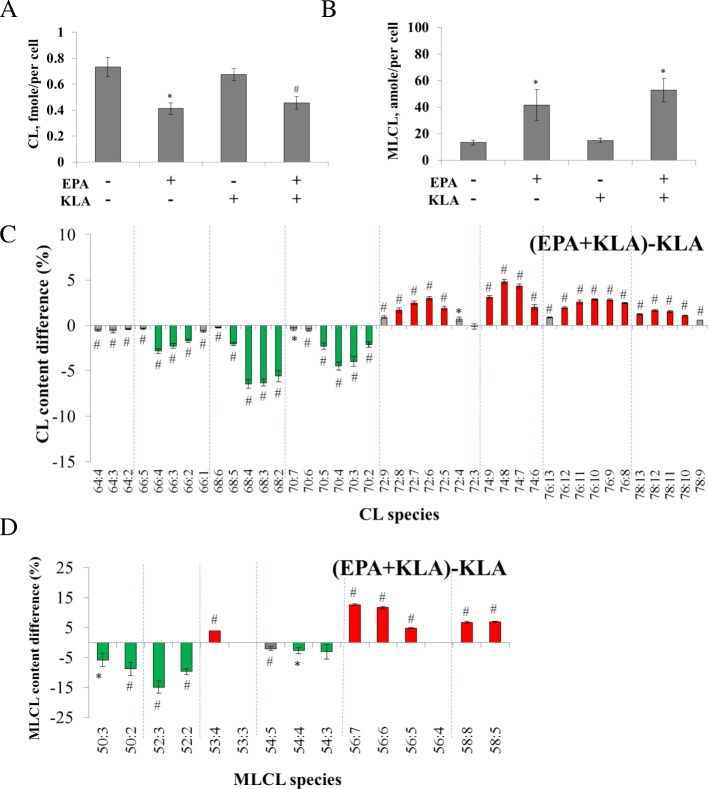
The change of amount and contents of CL and MLCL after EPA and KLA addition. RAW264.7 cells are stimulated by 100 μM EPA and 100 ng/ml KLA. The amount of CL (**a**) and MLCL (**b**) are quantified and and normalized via protein quantification. The CL and MLCL species difference between AA+KLA and KLA group are performed by direct deduction and shown in panel (**c**) and panel (**d**). Red represents the content difference more than 1 and 2.5% whereas green more than − 1 and − 2.5%, which is the one-fifth of the maximal difference, in CL and MLCL measurements, respectively. *p < 0.05, #*p* < 0.01

Similar results to the activated RAW264.7 cells were also observed with EPA treatment, in which EPA supplementation decreased 32% of CL, decreased short-chain CL, and increased long-chain CL. In MLCL analysis, EPA supplementation also induced 3-fold MLCL increase, decreased short-chain MLCL, and increased long-chain MLCL (Fig. 5). Without KLA activation, EPA supplementation showed similar trend of CL as the KLA-activated RAW264.7 cells (Additional file 3), indicating EPA supplementation induced CL remodeling overwhelmed the effects of KLA addition on CL desaturation. The MLCL/CL ratio increased 5.3-fold and 5.5-fold after EPA supplementation in active and inactive RAW264.7 cells.

### 20-carbon EPA and AA showed high CL incorporation efficiency

**Fig. 6:**
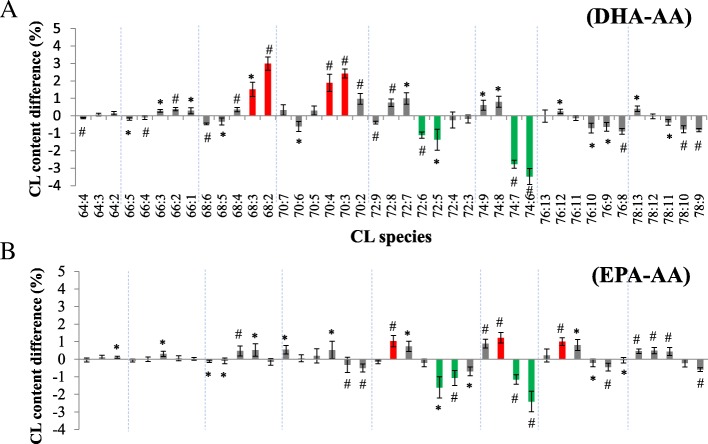
The comparisons of CL amount and content among DHA, EPA and AA supplementation following KLA activation. The differences are shown by (**a**) DHA and (**b**) EPA deducting AA. Red represents the content difference more than 1 and 2.5% and green represents more than − 1 and − 2.5% in CL and MLCL comparison, respectively. All color marked changes are the one-fifth of the maximal difference. *p < 0.05, #*p* < 0.01

AA (20:4; ω-6), EPA (20:5; ω-3) and DHA (22:6; ω-3) contain different chain lengths and double bonds. Therefore, we analyzed the differential incorporation or impact of these three types of PUFA on the species changes of CL. In the above experiments, PUFA supplementation induced an increase in MLCL/CL ratio and a shift to long-chain CL and MLCL contents. We compared the effect of ω-3 and ω-6 PUFA supplementation on CL acyl chain composition (Fig. 6). The results showed that in C68 and C70 groups, DHA induced less unsaturated CL (C68:3, C68:2, and C70:3) and C70:4 elevation compared to that with AA supplementation (Fig. 6a). Reversely, AA supplementation promotes the increase of the CL with higher acyl chain length, even though DHA per se contains higher chain length. This result indicated DHA has lower incorporation efficiency than AA.

In comparisons of EPA and AA effects, EPA induced long-chain unsaturated CL (C72:8, C74:8, and C75:11) elevation, whereas AA caused long-chain CL containing less DBs (C72:5, C72:4, C74:7, and C74:6) (Fig. 6b), which suggested that both EPA and AA contain identical chain length but different numbers of double bonds (DBs).

**Fig. 7:**
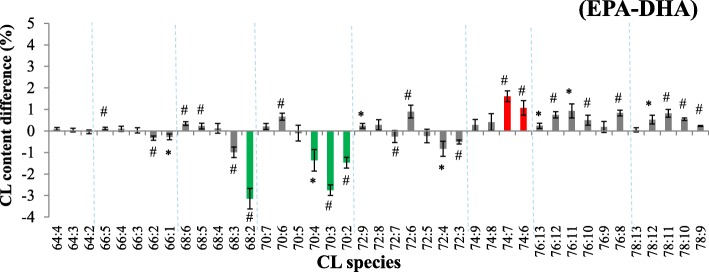
The comparison of CL amount and content between EPA and DHA supplementation following KLA activation. The differences are shown by EPA deducting DHA. Red represents the content difference more than 1 and 2.5% and green represents more than − 1 and − 2.5% in CL and MLCL comparison, respectively. All color marked changes are the one-fifth of the maximal difference. *p < 0.05, #*p* < 0.01

In the comparison of ω-3 PUFA, DHA induced increase of short-chain less unsaturated CL (C68:2, C70:3, and C70:2) and C70:4, whereas EPA elevated generally long-chain unsaturated CL (Fig. 7). This is a similar scenario as the comparisons between DHA and AA. This suggests 20-carbon EPA and AA exerting better incorporation efficiency into mitochondrial CL or higher impact to remodel CL than 22-carbon DHA.

## Discussion

The activation of RAW264.7 cells by KLA increased the saturation of CL [27], through the inhibition of desaturase. Further quantitation of each species of CL and MLCL revealed that less unsaturated CL and MLCL species increased in active macrophages, but the total quantity of CL and MLCL maintained constantly. The double bonds on the unsaturated fatty acyl chains of CL have high risk of oxidation and peroxidation. Macrophage activation and polarization has been shown to increase the intracellular reactive oxygen species (ROS) [28], which can attack the double bonds on CL. The increase of CL saturation reflects the resistance ability to oxidative stress, which suggested the cytoprotective role of less unsaturated CL [29]. By maintaining CL and MLCL at constant level, the active macrophage can prevent the damage from oxidative stress without drastically changing the membrane structure and function of mitochondria.

Several studies have shown that saturated fatty acids induce proinflammatory response through TLR4, by receptor dimerization and recruitment into lipid rafts [30–32]. In our experiments, we observed the increase of less unsaturated CL by KLA-stimulating TLR4, rather than supplying saturated fatty acids, which suggests that inhibition of the desaturase to increase the saturated fatty acid content in phospholipids is a mean to induce the inflammatory response. Downstream inflammatory signaling might be stimulated by less saturated fatty phospholipids, including CL. In addition, CL has been found to be associated with NLRP3 inflammasome activation, promoting the production of the proinflammatory cytokine IL-1β [18, 19].

We showed that PUFA supplementation decreased the amount of CL but increased the amount of MLCL; however, this effect was independent of KLA activation, which is because the effects of PUFA are massive and have overwhelmed the effect from KLA activation. CL decrease resulted from PUFA supplementation could be due to the decrease of CL biosynthesis. Alternatively, the increase of CL hydrolysis and remodeling can result in less CL and more MLCL. It has been shown that the expression of fatty acid transport protein-1 (FATP-1) reduces CL biosynthesis in HEK293 cells [33]. Another study showed that different *n*-3/*n*-6 ratio regulates FATP-1 mRNA expression [34]. Therefore, PUFA supplementation induced CL decrease in our results might be caused by the PUFA -induced FATP-1 expression to decrease the biosynthesis of CL.

The decrease in CL biosynthesis has been implied to play an important role in cytochrome *c* release in palmitate-induced cardiomyocyte apoptosis [35], decreased respiratory chain capacity, and increased ATP synthesis in human HepaRG cells [36]. However, the decrease of CL can also be a protective mechanism to minimize unsaturated CL and CL peroxidation. Indeed, another study showed that in CL synthase-knockdown HeLa cells, CL reduction had no impact on mitochondrial functions but increased the resistance to actinomycin D-, rotenone-, and gamma-irradiation-induced apoptosis [37]. However, CL decrease and MLCL accumulation, which were found in our study, have also been found in Barth syndrome patients and have been shown to link to tafazzin mutation [11, 38–41]. Thus, PUFA-induced CL decrease and MLCL elevation could suffer the similar functional defects of mitochondria.

In our study, PUFA addition increased long-chain CL and MLCL, whereas it decreased short-chain CL and MLCL. The increase of C74, C76, and C78 groups could be due to PUFA incorporation. However, in CL identification, these increased CL species are not necessarily all formed by the supplemented PUFA, indicating a more complex incorporation process. In a meta-analysis study, the author also suggested that DHA is incorporated into CL at the expense of linoleic acid at a level of up to 20% in the heart and 10% in the liver [5]. DHA, EPA, and AA supplementation may also affect linoleic acid metabolism, resulting in the change of linoleic acid-containing CL.

In the comparison of CL composition among DHA, EPA, and AA treatment, we showed that DHA generally decreases AA-containing CL, but predominant changes were found in the increased short-chain, less unsaturated CL and the decreased long-chain, eicosatrienoic acid-containing CL. It indicates that DHA supplementation not only facilitated the incorporation of DHA into CL, but more importantly also altered the remodeling of CL. Moreover, in the present study, we suggested another protective mechanism of DHA via CL compared with AA, i.e., elevation of less unsaturated CL, which is less sensitive to oxidation stress, and partly “neutralizes” the susceptibility of being oxidized due to large unsaturated CL elevation in supplementation. In MLCL analysis, DHA supplementation increased less unsaturated species and also the species containing AA and DHA, which could be the result of the integrated effect of DHA incorporation and replacement of AA.

DHA and EPA contain six and five DBs, which are sensitive to free radicals and cause lipid peroxidation; however, several reports supported the protective role of DHA and EPA by forming pre-resolving lipid mediators [2]. Unlike the 22-carbon DHA, EPA and AA both contain 20 carbon atoms, which both showed better incorporation efficiency than DHA, which maybe cause by the efficient conversion of the 20-carbon fatty acids to eicosanoids. Therefore, EPA supplementation can significantly increase the unsaturation of CL.

Macrophage activation significantly remodeled CL and shifted CL to saturated fatty acyl species, which could prevent the potential damage from ROS. Indeed, these mitochondrial phospholipid changes have been shown to be accompanied with the production of ROS and pro-inflammatory cytokines [42, 43]. Supplementation of AA, EPA and DHA significantly increased the MLCL/CL ratio of the activated macrophage, directly indicating an ongoing CL remodeling process. Interestingly, we observed the 22-carbon DHA (22:6) showed less CL remodeling efficiency than the 20-carbon EPA (20:5) and AA (20:4). Coincidentally, DHA has shown the strongest inhibitory effects on both ROS and RNS formation by LPS-stimulated macrophages, although EPA also significantly inhibited RNS production [44]. EPA and DHA also both displayed the differential anti-inflammatory potential through cytokine production in macrophage [43]. DHA was again showing higher potency to inhibit inflammation than EPA. EPA and DHA down-regulated the proinflammatory cytokine production of Interleukin IL-1β, IL-6 and tumor necrosis factor-α (TNF-α) secretion, and showed down-regulation of the cytokine production related gene expression of NF-κB (p65) and IκBα in the activated macrophage.

## Conclusions

We have shown that KLA activation induces decrease of unsaturated CL species and increase of less unsaturated CL species in RAW264.7 cells. Supplementation of AA, DHA, or EPA results in the decrease of CL, increase of MLCL, increase of long-chain species, and reduction of short-chain species in both CL and MLCL groups, regardless of KLA activation. 20-carbon AA and EPA have better incorporation efficiency; however, DHA can potentially become a potential competitor or neutralizer to AA.

## Additional files

Additional file 1: The changes of CL and MLCL species with AA supplementation in RAW 264.7 cell without KLA. CL (A) and MLCL (B) species are analyzed by LC-MS. In panel (A), red bar shows changes more than 1% and green bar shows changes more than − 1%, which is the one-fifth of maximal changes. In panel (B), red bar shows changes more than 2.5% and green bar shows changes more than − 2.5%, which is the one-fifth of maximal changes. (DOCX 37 kb)

Additional file 2: The changes of CL and MLCL species with DHA supplementation in RAW 264.7 cell without KLA. In panel (A), CL species with red bar shows changes more than 1% and green bar shows changes more than − 1%, which is the one-fifth of maximal changes. In panel (B), MLCL species red bar shows changes more than 2.5% and green bar shows changes more than − 2.5%, which is the one-fifth of maximal changes. (DOCX 38 kb)

Additional file 3: The changes of CL and MLCL species with EPA supplementation in RAW 264.7 cell without KLA. In panel (A), CL species with red bar shows changes more than 1% and green bar shows changes more than − 1%, which is the one-fifth of maximal changes. In panel (B), MLCL species red bar shows changes more than 2.5% and green bar shows changes more than − 2.5%, which is the one-fifth of maximal changes. (DOCX 38 kb)

## Abbreviations

AA: Omega-6 arachidonic acid
CL: Cardiolipin
DHA: Omega-3 docosahexaenoic acid
EPA: Omega-3 eicosapentaenoic acid
KLA: KdO_2_-lipid A
LC-MS: Liquid chromatography-mass spectrometry
MLCL: Monolysocardiolipin
ROS: Reactive oxygen species
XIC: Extract ion current
