# Supplementary material for: Omega-3 and omega-6 fatty acid differentially impact cardiolipin remodeling in activated macrophage
Source: Lipids Health Dis. 2018 Aug 28;17:201. doi: 10.1186/s12944-018-0845-y (PMC6114728; doi:10.1186/s12944-018-0845-y)
Supplement: Supplementary file 1 — The changes of CL and MLCL species with AA supplementation in RAW 264.7 cell without KLA. CL (A) and MLCL (B) species are analyzed by LC-MS. In panel (A), red bar shows changes more than 1% and green bar shows changes more than − 1%, which is the one-fifth of maximal changes. In panel (B), red bar shows changes more than 2.5% and green bar shows changes more than − 2.5%, which is the one-fifth of maximal changes. (DOCX 37 kb) [file 12944_2018_845_MOESM1_ESM.docx]

**Title: Omega-3 and Omega-6 Fatty Acid Differentially Impact Cardiolipin Remodeling in Activated Macrophage**

**Authors: Wan-Hsin Chang^1#^, Hsiu-Chi Ting^1#^, Wei-Wei Chen^1^, Jui-Fen Chan^1^ and Yuan-Hao Howard Hsu^1,2^***

^#^These authors contributed equally to this work.

**Affiliations:**^1^Department of Chemistry, ^2^Life Science Research Center, Tunghai University, Taichung, Taiwan

Address: No.1727, Sec4, Taiwan Boulevard, Xitun District, Taichung 40704, Taiwan R.O.C

*Corresponding author: YHH

E-mail: howardhsu@thu.edu.tw

Additional file 1

Additional file 1 The changes of CL and MLCL species with AA supplementation in RAW 264.7 cell without KLA. CL (A) and MLCL (B) species are analyzed by LC-MS. In panel (A), red bar shows changes more than 1% and green bar shows changes more than -1%, which is the one-fifth of maximal changes. In panel (B), red bar shows changes more than 2.5% and green bar shows changes more than -2.5%, which is the one-fifth of maximal changes.
