# Supplementary material for: Omega-3 and omega-6 fatty acid differentially impact cardiolipin remodeling in activated macrophage
Source: Lipids Health Dis. 2018 Aug 28;17:201. doi: 10.1186/s12944-018-0845-y (PMC6114728; doi:10.1186/s12944-018-0845-y)
Supplement: Supplementary file 2 — The changes of CL and MLCL species with DHA supplementation in RAW 264.7 cell without KLA. In panel (A), CL species with red bar shows changes more than 1% and green bar shows changes more than − 1%, which is the one-fifth of maximal changes. In panel (B), MLCL species red bar shows changes more than 2.5% and green bar shows changes more than − 2.5%, which is the one-fifth of maximal changes. (DOCX 38 kb) [file 12944_2018_845_MOESM2_ESM.docx]

Additional file 2 The changes of CL and MLCL species with DHA supplementation in RAW 264.7 cell without KLA. In panel (A), CL species with red bar shows changes more than 1% and green bar shows changes more than -1%, which is the one-fifth of maximal changes. In panel (B), MLCL species red bar shows changes more than 2.5% and green bar shows changes more than -2.5%, which is the one-fifth of maximal changes.
